# Navigating the Human Side of AI: A Socio-Technical Model of Employee Attitudes Toward Algorithmic Recruitment

**DOI:** 10.3390/bs16091708

**Published:** 2026-09-21

**Authors:** Hasan Beyari

**Affiliations:** Department of Administrative and Financial Sciences, Applied College, Umm Al-Qura University, Makkah 24382, Saudi Arabia; hmbeyari@uqu.edu.sa

**Keywords:** artificial intelligence (AI), employee perception, recruitment and selection, organisational support, technostress, AI integration clarity

## Abstract

This study investigates employee perceptions of artificial intelligence (AI) in the field of recruitment and selection in Saudi Arabian companies. While the use of recruitment systems based on AI is growing, their acceptance by organisations is influenced by whether employees consider these systems to be useful, transparent, and fair, and how this affects their professional judgement. This study is based on the Unified Theory of Acceptance and Use of Technology (UTAUT) and Socio-Technical Theory (STT) and explores a two-path explanatory model. Perceived usefulness is linked to perceived AI effectiveness in the AI-related pathway and is associated with transparency of AI, trust in AI systems, and organisational support for AI use. On the HR pathway, there is a negative association between technostress and HR support and readiness, while clarity of integration of AI is positively associated with HR support and readiness. The sample comprised 450 purposively selected respondents from Jeddah, Riyadh and Dammam, and data were analysed using Structural Equation Modelling (SEM) in AMOS. The perceived effectiveness of AI and HR’s support/readiness for AI was positively and significantly correlated with employee perceptions of AI in recruitment and selection. The relationships of these data should be interpreted as structural relationships; since the data are cross-sectional, they cannot be interpreted as causation or mediation. Further methodological issues include common method bias since all constructs were assessed via self-report and in the same questionnaire.

## 1. Introduction

Artificial intelligence (AI) has revolutionised the recruitment and selection process in various industries, particularly in digitally transforming nations like Saudi Arabia. AI is becoming increasingly important in the realm of human resource management (HRM), where it is used in résumé screening and automated shortlisting, candidate matching, predictive recruitment analytics, and even for communication through chatbots. In this regard, 85% of the HR professionals in the Kingdom reported that they are experiencing significant shifts in HR practices due to technological integration, consistent with the overall objective of Vision 2030 (Alsalman, 2025; Saudi Vision 2030, n.d.; Saudi Data & AI Authority, n.d.). New studies indicate that AI is transforming the recruitment industry by improving the speed, consistency and data-driven support of the recruitment decision-making process (Safshekan et al., 2026). Simultaneously, staff and job seekers can feel a lack of transparency, personalization, fairness, and/or ability to challenge (Tursunbayeva et al., 2025) in the systems they are using for recruitment. Whilst AI can improve the efficiency of recruitment, there are concerns regarding fairness, depersonalisation, governance and people’s decreased influence over hiring decisions (Aleisa et al., 2023; Alsaedi et al., 2024). Thus, the sustainability of the use of AI in the recruitment process also relies on employees’ perceptions of systems being legitimate, useful and compatible with their job.

In spite of the increasing number of studies that consider accuracy, algorithmic bias and system optimisation in AI-supported recruitment and selection, empirical and theoretical gaps remain in explaining employee perceptions. Current research primarily focuses on how technology affects staff experiences as they work with AI and not on the consequences of such technology. This creates a barrier to adoption not only because of the efficiency or predictive quality of AI recruitment but also because of the socio-organisational challenges related to trust, transparency, technostress, HR support, role clarity and threat of professionalisation. However, as indicated in the algorithmic HRM literature, AI can also affect the degree of human autonomy, accountability, and human agency when working on a digital platform (Chen, 2026; Köchling et al., 2025), which also explains the lower satisfaction and weak perceptions of fairness observed when human involvement is reduced in career decisions (Köchling et al., 2025). Equally, AI-induced technostress highlights that worker responses to intelligent systems are based on the technological needs of the system and social aspects linked with the implementation of intelligent systems (Jin et al., 2026). A broader perspective is, thus, required that includes trust, technostress, in addition to organisational support, clarity on how to integrate AI, perceived effectiveness of the AI used, HR support and perceived threats to professional autonomy. This study is based on the Unified Theory of Acceptance and Use of Technology (UTAUT) and the Socio-Technical Theory (STT), which allows for the study of these technological and organisational factors in combination (Venkatesh et al., 2003; Trist & Bamforth, 1951), which enables technology acceptance factors and HR-related socio-technical conditions to be analysed in a single structural model. This study employs survey data collected from Saudi Arabia and utilises Structural Equation Modelling (SEM) using AMOS to explore the interconnections among the factors affecting employee perception of AI in recruitment and selection.

## 2. Literature Review

### 2.1. AI Components

#### 2.1.1. Transparency of AI

Transparency can significantly impact employee reactions towards AI solutions, especially in the HR domain. Employees are more likely to assess the AI system and the results of it when they understand what information it is using, how it generates recommendations and how it outputs that information is going to be used to make recruitment decisions. Workers tend to feel more comfortable and less fearful of concealed bias or discriminatory actions when they are exposed to transparent AI systems, which can alleviate anxiety (Employees’ appraisals and trust of artificial intelligences’ transparency and opacity, Yu et al., 2023). Moreover, despite the fact that the complexity of the system’s processes may make workers feel disposable at first, open communication has been shown to increase the efficacy of AI tools with regard to workers (Artificial intelligence decision-making transparency and employees’ trust: The parallel multiple mediating effect of effectiveness and discomfort, Yu & Li, 2022). In addition, recent studies in the field of AI-HRM highlight the correlation between transparency and fairness, explainability, and preservation of human agency, especially when AI-driven systems impact recruitment and selection processes (Chen, 2026; Naoum et al., 2026). In this sense, transparency is not merely a technical aspect but a key component to creating trust and compliance among employees regarding the aims of the technology of the organisation.

#### 2.1.2. AI Systems Trust

One important aspect that can determine uptake of AI systems among employees is trust, particularly in situations that entail the use of discretion such as hiring. The technical soundness of AI tools is questioned when employees do not believe that they are fair, accurate, and/or moral. Trust is regarded as a key enabler of AI adoption, and lack of trust hinders the willingness to adopt and accept AI systems (Afroogh et al., 2024). Trust should be used initially since the initial impressions of employees towards an AI system can influence their readiness to further interact with this system, particularly when the upper management is able to express themselves properly and show confidence in the technology (Y. Xu et al., 2024). Recruitment AI also relies on the ability of the employees to comprehend the working mechanism, doubt its results and count on proper human control (Naoum et al., 2026). As a result, organisational investment in technology and sincere and transparent human practices regarding AI incorporation will be needed to generate trust in AI.

#### 2.1.3. HR Organisational Support

The level of HR department-specific support is most effective for maximising employee acceptance of AI implementation. The presence of support systems, in the form of AI training modules, codes of ethics, and bridges of communication, eliminates any type of uncertainty and builds confidence. The proposed indicators of institutional commitment to the responsible use of AI are symbolic leadership and visible management commitment (C. Hu et al., 2025). Open communication at the organisational level may also assist in decreasing the emotional burden of the felt threat of automation and prepare employees in modifying their roles in line with technological changes (R. B. Soomro et al., 2025). The latest AI-HRM studies underline that organisational support should assist employees to interact with AI in terms of training, human supervision and open governance mechanisms (Safshekan et al., 2026). This kind of help can decrease doubt and offer practical and psychological confidence in the shift towards AI-powered work.

#### 2.1.4. Perceived Usefulness of AI

The perceived usefulness of AI systems in recruitment processes is an important predictor of evaluation. The degree of volitional acceptance of AI by staff is associated with the extent of value they see in using AI to improve job performance (through decision making, efficiency, or time-saving) (Almeida et al., 2025). Across various industries, perception of usefulness plays a key role in determining the acceptance of technology, and it could be far more significant than other factors like ease of use or familiarity (Ibrahim et al., 2025). AI can enhance candidate matching, recruitment efficiency and decision support and boost employees’ perception of its practicality in HRM (Safshekan et al., 2026). Characteristics like reduced recruitment cycles and precise matching are also usually linked to the utilization of recruiting tools with AI capacities (Aleisa et al., 2023). This increases the likelihood of employee acceptance of AI systems in the workplace when they are able to see the value in using AI within their work.

#### 2.1.5. Perceived AI Effectiveness

Perceived AI effectiveness is the employees’ judgment of how effective the AI recruitment systems are in enhancing work efficiency, decision quality, goal alignment and general recruitment outcomes. The perceived usefulness is not the only factor that affects this assessment but also the reliability of the system, transparency, trust, and the support of the organisation. AI can assist HR in reducing time to hire, enhancing screening consistency, better accuracy in candidate matching and creating outputs that HR personnel can comprehend and utilize (Safshekan et al., 2026). Nevertheless, good recruitment AI must also be unbiased, clarifiable and in harmony with human-based decision making (Naoum et al., 2026). Thus, perceived AI effectiveness is a potential appropriate bridge between technical factors of AI and the overall attitudes of employees towards AI in recruitment.

### 2.2. HR Components

#### 2.2.1. Technostress

Technostress is a crucial psychological component in employees’ interactions with AI recruitment systems. The stress associated with adapting to new and continuously evolving technologies can contribute to anxiety, disengagement, and resistance. Technostress in HR contexts can decrease the readiness of employees to work with AI and augment psychological stress (Chang et al., 2024) because judgement, precision and flexibility are essential in HR contexts. The technological stress created by AI can also be seen as either a challenge or an impediment, depending on the perception of employees about the technological change as being manageable or disruptive (Jin et al., 2026). It is also possible to influence the reactions of employees to intelligent technologies through psychological and emotional processes, such as identity-related appraisals and emotional exhaustion (Human–Robot Collaboration and Organizational Citizenship Behavior: The Psychological and Emotional Mediating Pathways, Duan et al., 2026a). Thus, managing technostress is important for sustaining constructive employee engagement with AI.

#### 2.2.2. Perceived Threat to Professional Judgement

How employees perceive recruitment technology is greatly influenced by their fear of a technologically assisted workplace. Employees are afraid that their human expertise will be devalued by the AI system, and that a large part of their work consists of making judgements and evaluations that are too nuanced for the technology. Once employees perceive that their work will be undermined by the technology, their trust in AI and the acceptance of AI systems will plummet (Köchling et al., 2025). This is especially the case for employees that have strong attachment to their work, have discretion in their work, or perform mainly evaluative work (Ackerhans et al., 2025). This includes employees who work in the healthcare sector, the property sector, and other sectors in which their work is greatly influenced by their identity, the discretion that they have in their work, and the numerous evaluations that they have to make on a daily basis.

#### 2.2.3. Clarity of AI Integration

Clarity of AI integration refers to how clearly organisations communicate how AI is being integrated, why it is being introduced, and the limits of its use. Transparency of AI integration means that organisations need to justify the introduction of AI by stating why it is being implemented, the tasks it will carry out and the expected results. When employees lack awareness of the purpose or value addition of systems that leave the comfortable way of working, they may become resistant to the digital transformation (Gong et al., 2024). When technology is being introduced that is different from what the employees are used to, they can be reluctant to adopt it (Gong et al., 2024). Having a clear implementation roadmap and inter-departmental communication can help minimize uncertainty over technological change (Khan et al., 2024). It is also essential that the employees have a clarity of delineation of responsibilities between human decision makers and AI systems, especially in terms of accuracy, fairness and accountability (Naoum et al., 2026). Role clarity, then, is a key factor that influences employees’ intentions to endorse technology-enabled organisational change (Wu et al., 2025). Thus, the clarity of AI integration is an important organisational condition influencing how employees interpret AI-related change.

#### 2.2.4. HR Support and Readiness

HR support and readiness describe the ability of HR structures, policies and staff to support employees in the context of AI implementation. This includes the process of educating users, offering communication, procedural support, and emotional support and, most importantly, a clear process of dealing with concerns regarding the use of AI. As increasingly advanced AI systems are introduced into our social environment and into organisations, it is important that employees can make sense of the decisions made by algorithms as well as of human intervention in these systems (Chen, 2026; Horodyski, 2023). Moreover, employees must be able to manage this transition to the new AI environment responsibly, where HR support is critical in cases where technostress and negative perceptions of using AI in the recruitment process bring employees’ trust in AI down. When organisations are prepared for AI, HR can make a significant contribution to a positive mindset for the staff, making the acceptance of AI an organisational change rather than an imposed technological change.

### 2.3. Perception of AI by Employees

The influence of technological change on organisational culture, employee engagement, and trust between employees and AI systems is important to comprehend, and employee attitudes can be a key indicator. Employee attitudes affect adoption and are closely interrelated with perceptions of the fairness, transparency, and utility of AI systems. In cases where employees feel that AI is robbing them of their power or violating procedural justice, they react by distrusting or fighting AI (Cai et al., 2024). Conversely, employee attitudes towards AI were found to be more positive in organisations that implemented participatory approaches, including involvement of staff in the implementation of the AI system and articulation of the anticipated benefits (Martín-Hernández, 2023). Recent research findings of AI-HRM also point to the fact that the attitudes towards AI are conditioned by the balance between system functionality, fairness, explainability, governance, and human-centric implementation (Naoum et al., 2026; Safshekan et al., 2026). Employee attitudes are not uniform but are negotiated and constructed socially in response to organisational climate and leadership cues.

### 2.4. Synthesis and Literature Gap

The literature indicates that the factors related to AI and HR are the two that influence the employees’ perceptions of AI in recruitment. On the one hand, factors of technical value and legitimacy, such as transparency, trust, organisational support and perceived usefulness, reflect the technical value and legitimacy of AI in recruitment; on the other hand, factors of socio-technical context in which AI is applied, such as technostress, perceived threat to professional judgement, integration clarity and HR support, reflect the socio-technical context in which AI is applied. The theory of technology acceptance research involves usefulness and facilitating conditions, and through this Socio-Technical Theory, it has been explained that there is interaction between technological and social systems in the workplace (Venkatesh et al., 2003; Trist & Bamforth, 1951). Fairness, transparency, human agency and governance are also issues that are pointed to in the recent AI-HRM research with regard to algorithmic HRM (Naoum et al., 2026; Chen, 2026). Based on this, this study postulates two paths of explanation: (1) AI-related antecedents to perceived AI effectiveness and (2) HR-related antecedents to HR support and readiness, which in turn relate to employee perceptions of AI. It reflects findings that AI can create “resource-enhancing” and “resource-straining” assessments, when employees interact with it (AI collaboration and quality of work life: a dual perspective of hotel employees’ perceptions, Duan et al., 2026b). This combined perspective provides the basis for the dual-path model tested in this study.

## 3. Theoretical and Conceptual Modelling

### 3.1. Unified Theory of Acceptance and Use of Technology (UTAUT)

The Unified Theory of Acceptance and Use of Technology (UTAUT) can be used to describe and explain employee acceptance and use of new technology in an organisational context. UTAUT merges eight models already known that have been used to explain technology acceptance and use. The four constructs used in this work, namely performance expectancy, effort expectancy, social influence and facilitating conditions, can be used to explain technology use and behavioural intention in organisational settings (Venkatesh et al., 2003; Said et al., 2024). Perceived usefulness of AI in this study is very similar to the performance expectancy as it addresses employees’ beliefs that AI will enhance efficiency, accuracy and decision support. Facilitating conditions involve the organizational resources which support the employees to incorporate technology in their work, such as training, guidance, communication, and implementation support (Dwivedi et al., 2019). The other key consideration in AI is trust and transparency, as staff must be confident in the reliability, explainability, fairness and accountability of AI systems before accepting its recommendations (Naoum et al., 2026). Therefore, UTAUT serves as a theoretical perspective to investigate the relationship between perceived AI effectiveness and the constructs of transparency, trust, usefulness and organisational support.

### 3.2. Socio-Technical Theory (STT)

The Socio-Technical Theory (STT) complements the understanding of UTAUT and its explanation of the relationship between technological systems and the social systems in which work is organised. STT was developed to describe how a workplace is impacted during technological change; it has been argued that technological innovation should be seen as a relationship between technological systems and social systems, not just technology itself (Emery & Trist, 1960). This theory is relevant for recruitment in the context of artificial intelligence, as it is not only relevant to the automation of tasks but also to the transformation of role expectations, professional autonomy, psychological stress and the role of human judgement in HR work. In this study, technostress and perceived threat to professional judgement are conceptualised as the social tensions generated when AI tools disrupt existing recruitment processes and the role identities of employees (Trist & Bamforth, 1951). In addition, recent socio-technical research reveals that technostress from AI can impact employees via challenge- and hindrance-related pathways, depending on the perceived manageability and support of the technological change (Jin et al., 2026). Clarity of AI integration is, thus, considered as a structural condition that can mitigate uncertainty by providing fundamental purpose, scope and boundaries for the use of AI. STT allows us to analyse the HR support/readiness pathway, as it demonstrates the links between technostress, questions regarding the professional judgement and clarity of the integration of AI and employees’ experiences of AI implementation.

### 3.3. Conceptual Model

**Figure 1:**
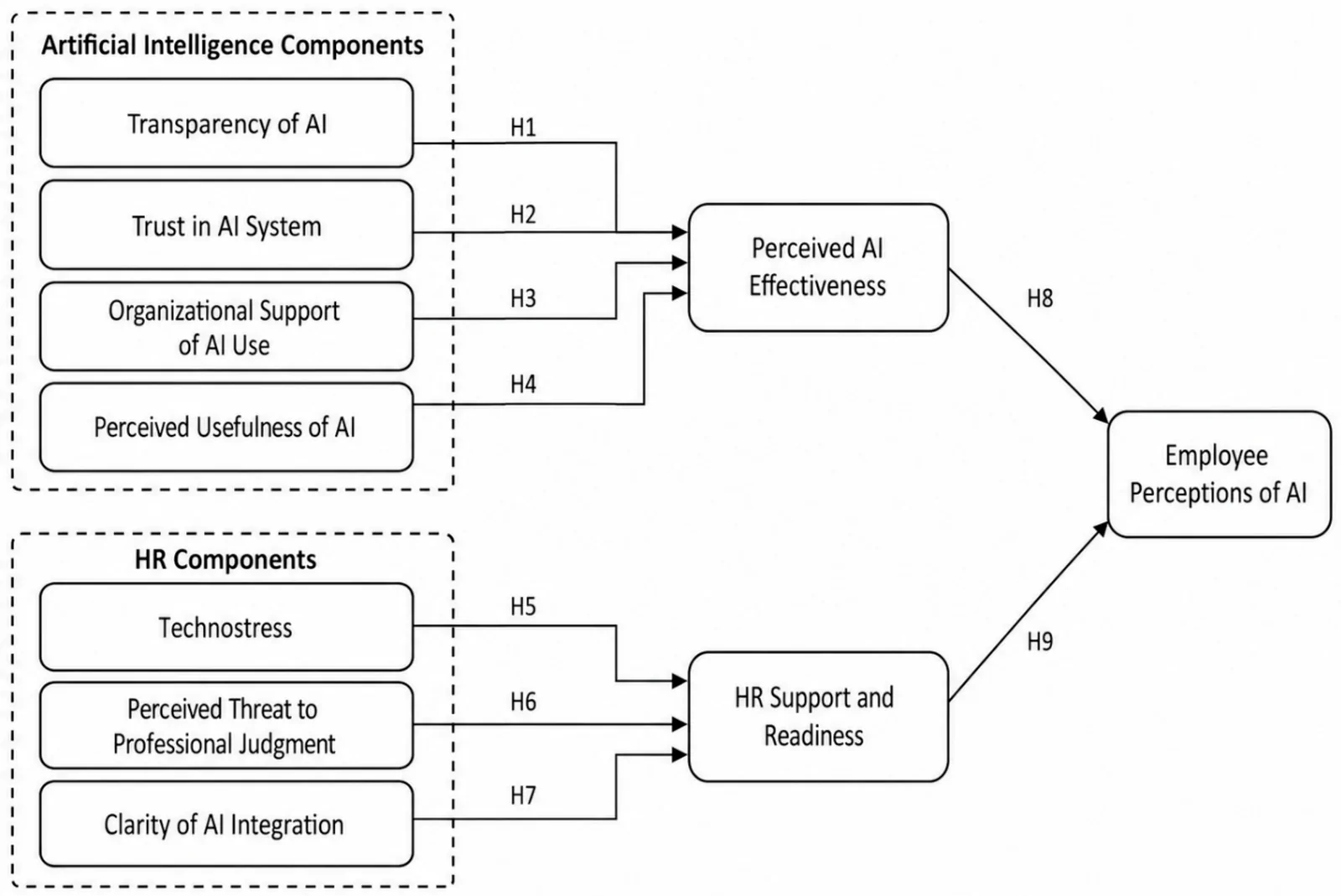
Conceptual framework.

In the conceptual model depicted in Figure 1, both technological and socio-technical viewpoints were integrated into one conceptual model of employees’ perceptions of the use of AI in recruitment and selection. It includes two explanatory pathways—that of the effectiveness of AI and another related to HR support/readiness. To echo technology acceptance explanations of performance value and facilitating conditions (Venkatesh et al., 2003), transparency of AI, trust in AI systems, organisational support for AI use and perceived usefulness of AI are identified as antecedents of the perceived effectiveness of AI. Based on the socio-technical interaction (between technology, work design and employee experience) (Trist & Bamforth, 1951; Pasmore, 1985), technostress and perceived threat of professional judgement and clarity of AI integration are identified as antecedents of HR support and readiness. The perceived effectiveness of AI and the HR support/readiness are used as intermediate explanatory variables to connect these antecedents with the employee perceptions of AI but do not imply statistical mediation.

## 4. Hypothesis Development

### 4.1. H1: Transparency of AI and Perceived AI Effectiveness

An important factor that defines how employees cognitively and emotionally react to artificial intelligence (AI) systems in the workplace is transparency. The more employees understand how the implemented AI systems operate, what information these systems are based on, how they analyse such information, and how decisions are made, the more likely they are to feel confident, transparent, and in control of using these systems. Being more transparent can enhance trust and make employees less scared of AI by simplifying its processes (Employees’ appraisals and trust of artificial intelligences’ transparency and opacity, Yu et al., 2023). Assessments of AI effectiveness and trust may also be reinforced by perceived transparency, but still, employees might feel a certain level of discomfort when engaging with AI systems (Artificial intelligence decision-making transparency and employees’ trust: The parallel multiple mediating effect of effectiveness and discomfort, Yu & Li, 2022). Transparency is also of great significance in AI-based recruitment since employees and applicants should comprehend how data are handled and how recommendations are drawn to be able to assess the accuracy and efficiency of the system (Naoum et al., 2026; Chen, 2026). Transparency is, thus, anticipated to enhance the perceived AI effectiveness by rendering recruitment technologies more comprehensible, agreeable and helpful to HR decision support.

**H1.**  *Transparency of AI is significantly and positively associated with perceived AI effectiveness.*

### 4.2. H2: Trust in AI Systems and Perceived AI Effectiveness

Trust has been widely considered the most critical determinant influencing user attitudes towards intelligent systems. Without trust as the foundation, users will not adopt and interact positively with AI technologies, regardless of how well they perform their objective functions. Researchers state that trust may aid in adhering to and using AI, but distrust may deter interaction with intelligent systems (Afroogh et al., 2024). First, trust is particularly crucial in organisational contexts as it is connected to the readiness of employees to embrace AI-related change (Y. Xu et al., 2024). Trust in AI may also be enhanced by the confidence of employees towards organisational leaders as it may indicate that the systems are managed and adequately supported (Y. Xu et al., 2024). In the hiring process, employees are not likely to consider an AI system effective when they believe that it is unfair, unreliable or irresponsible (Kovács & Horváth, 2025; Naoum et al., 2026). It is anticipated that perceived AI effectiveness will enhance employees’ trust in AI in making recruitment decisions in a responsible and consistent manner.

**H2.**  *Trust in AI systems is significantly and positively associated with perceived AI effectiveness.*

### 4.3. H3: Organisational Support for AI Use and Perceived AI Effectiveness

Organisational support impacts employee experience and engagement with technological innovation, or AI in this case. Employees can be more amenable to AI through organisational support, which can offer management support, training and expectations regarding its use. Symbolic support and structural support can, as well, diminish the perception of threat among employees and enhance their readiness to accept AI systems (C. Hu et al., 2025). This is in line with the findings of (G. Xu et al., 2023), who reported that perceived organisational support mediated the relationship between emotional exhaustion and stress related to the use of AI, thus serving to relieve employees of perceptual strain. The practical application of AI in HRM is dependent on organisational support, since employees must be trained and advised to implement and apply an AI system to ensure that its potential is converted into actual HR practice (Safshekan et al., 2026). Therefore, it is expected that organisational support has a positive impact on employees’ perceptions of the effectiveness of AI, as employees are more prepared to use, understand, and assess recruitment AI systems.

**H3.**  *Organisational support for AI use is significantly and positively associated with perceived AI effectiveness.*

### 4.4. H4: Perceived Usefulness of AI and Perceived AI Effectiveness

Perceived usefulness—the extent that an employee thinks using a given system will increase their job performance—is a key determinant of employee perception and acceptance of AI technologies. This construct is at the heart of the Technology Acceptance Model (TAM) and can be examined empirically in various work contexts. According to (Almeida et al., 2025) and (Hewage, 2023), recruiters tended to use AI tools more when they thought that these tools were productive and efficient in terms of personal and task performance. Perceived usefulness may be more powerful in encouraging positive attitudes towards AI than perceived ease of use. The willingness of staff to embrace AI increases when they feel that it will enhance their job and have a definite practical benefit (Ibrahim et al., 2025). In recruitment and selection, usefulness is measured in terms of perception that AI can enhance the efficiency of screening, matching of candidates, decision quality and overall effectiveness of the HR processes (Safshekan et al., 2026). Therefore, perceived usefulness is expected to enhance the perceptions of employees towards AI systems as an efficient recruitment support tool.

**H4.**  *Perceived usefulness of AI is significantly and positively associated with perceived AI effectiveness.*

### 4.5. H5: Technostress and HR Support and Readiness

Technostress is stress that occurs when new technologies are implemented abruptly. It has become a relevant feature of employee attitudes towards the application of artificial intelligence (AI) in the workplace. As AI becomes increasingly popular in the workplace, replaces traditional practices and brings new approaches to decision making, employees may experience psychological and performance stress during the implementation process, which may change their attitudes towards AI systems in general (Magdalenić & Luić, 2025; Madanchian, 2024). Technostress linked to AI is usually characterised by a sense of threat to the self and occurs when individuals feel unable to engage with the AI tools provided and feel anxiety, mistrust, and hesitation to use them (Golgeci et al., 2025). Using empirical evidence, (Kim & Lee, 2024) confirmed the correlation between job stress and AI adoption and how it may lead to burnout, which can be moderated by individual traits such as self-efficacy. Technostress can be a challenge or a hindrance, depending on employees’ perception of the development of AI as manageable or as another source of stress (Jin et al., 2026). If employees feel that AI is causing them stress, overload or anxiety, they may not consider HR to be ready to support AI implementation. Technostress is expected to be negatively associated with HR support and readiness; therefore, as technostress increases, HR support and readiness will decrease.

**H5.**  *Technostress is significantly and negatively associated with HR support and readiness.*

### 4.6. H6: Perceived Threat to Professional Judgement and HR Support and Readiness

The use of AI can also cause existential anxieties among employees, especially when they feel threatened by AI for taking away their job competency or ability to make decisions. When employees feel that AI is taking over and supplanting their own abilities and decision making, it can have a negative impact. This might lead to a decrease in the perceived value of AI and reluctance to use it due to the perceived threats to identity and autonomy (Golgeci et al., 2025). This is especially the case for professions that are based on judgement. In their study on the interaction between healthcare and AI, (Ackerhans et al., 2025) concluded that clinicians who felt that their profession was devalued by the use of AI showed reduced trust and increased resistance. Moreover, algorithmic HRM changes the allocation of human agency and responsibility within the company, especially when decisions were previously made by humans with the help of automated tools (Chen, 2026). Hence, perceived threat to professional judgement is likely to undermine HR support and readiness, since employees might believe that the introduction of AI poses a risk to the security of their jobs, discretion, and professional involvement.

**H6.**  *Perceived threat to professional judgement is significantly and negatively associated with HR support and readiness.*

### 4.7. H7: Clarity of AI Integration and HR Support and Readiness

By communicating about the implementation of AI, individuals will be able to influence employee attitudes and the relationship between technological changes and their organisation’s goals. Organisations that make it clear to employees what the purpose of AI is, the potential benefits and how it will fit into their current work practices are more likely to have employees understand and accept AI (Shahiduzzaman, 2025; Gong et al., 2024). Lack of understanding of the uses of AI can lead to resistance and a lack of trust (Mwita & Kitole, 2025). When implemented well, it can enhance engagement, foster a sense of security and decrease uncertainty (Kheterpal et al., 2024). Notably, there is greater acceptance of AI within organisations when they communicate how AI will not just replace their jobs but augment them (Khan et al., 2024). It is the clarity of AI integration, however, that should enhance HR support and preparedness as it allows the employees to see why AI is being implemented, what decisions are left to be made by humans and how AI suggestions are to be viewed.

**H7.**  *Clarity of AI integration is significantly and positively associated with HR support and readiness.*

### 4.8. H8: Perceived AI Effectiveness and Employee Perceptions of AI

Perceived AI effectiveness is the rating that employees give an AI system regarding how it affects the efficiency of recruitment, the quality of the decisions made and consistency, and the alignment of the AI system with HR goals. An AI system that is trustworthy, open, and can aid in recruitment decisions is more likely to have a positive effect on how employees view AI. Recent research indicates that AI may be useful in different HR functions, such as recruitment, training, performance management, compensation, and retention, as long as its outputs are relevant to the organisational users and are related to HR activities (Safshekan et al., 2026). Perceived effectiveness is relevant in the context of recruitment since AI must enhance the results of recruitment without interfering with the aspects of fairness, explainability, or professional responsibility (Naoum et al., 2026). Those employees who consider AI to be effective will consequently have more favourable attitudes towards its application in hiring.

**H8.**  *Perceived AI effectiveness is significantly and positively associated with employee perceptions of AI.*

### 4.9. H9: HR Support and Readiness and Employee Perceptions of AI

HR support and readiness describe the extent to which HR are involved in and ready to lead employees in the AI implementation process. To have a positive impact on employee attitudes towards AI, HR should make sure that employees feel safe by maintaining a human touch, emotionally supported, and well-informed about the new procedures. Following implementation, technostress and resistance can be caused by the implementation of AI, as well as uncertainty and threats to their professional identity. HR support, such as providing understanding and adaptation, can be effective to help employees understand and adapt to these changes (Jin et al., 2026). Responsible governance is also crucial as employees must trust in the fairness and transparency of AI systems and that they are designed to ensure a level of human agency in decisions made in the workplace (Chen, 2026). Therefore, HR assistance and preparedness are likely to improve the perceptions of employees by making AI-based hiring be perceived as more relatable, supported, and career-friendly.

**H9.**  *HR support and readiness are significantly and positively associated with employee perceptions of AI.*

## 5. Research Methodology

### 5.1. Research Design

This study used a quantitative approach. A cross-sectional study design was employed using a structured survey to investigate the factors influencing employee attitudes towards artificial intelligence (AI) in recruitment and selection processes. A quantitative design was suitable as the aim was to explore measurable relationships between predefined latent constructs, and questions were asked with standardised responses, which could be compared between respondents. Given this study’s latent variables (transparency of AI, trust, perceived usefulness, technostress, organisational support, clarity of AI integration and perceived threat to professional judgement), a quantitative cross-sectional design was suitable as it aims to explore patterns of association between these variables. The snapshot in the design helped to capture the attitudes and perceptions of the employees at one moment, which made it possible to make no claim of temporal or experimental causality regarding the proposed relationships but to analyse them statistically (Wang & Cheng, 2020). A five-point Likert scale (Strongly Disagree, Disagree, Undecided, Agree and Strongly Agree) was used to standardise responses and enable comparison across constructs. This aligned with the study goal of assessing attitudes, perceptions, and evaluative responses about the use of AI in recruitment through structured scale items.

Structural Equation Modelling (SEM) was selected because it allows relationships among multiple latent variables to be estimated simultaneously (Kline, 2023). Due to the conceptual model used, which has multiple predictors (both AI and HR-related) as well as two mediators (perceived AI effectiveness and HR support/readiness) and employee perceptions of AI as an outcome, SEM was deemed to be the appropriate test. AMOS 28 was used to measure the model, the fit of the model and the hypothesised paths. The measurement model was tested prior to the structural model (i.e., following the usual two-step SEM method; (Kline, 2023)). This study was cross-sectional, so the results were viewed as cross-sectional relationships or structural relationships (Wang & Cheng, 2020).

### 5.2. Sample and Data Collection

The respondents were employees from organisations with the experience of utilising AI tools in recruitment or in the process of using them. The respondents were selected using a random sampling technique, and the sample size was 450, where three cities which are economic and technological centres of Saudi Arabia, Jeddah, Dammam and Riyadh, were sampled. The respondents had to have first-hand experience with or be familiar with the use of AI in recruitment (not the general population). Purposive sampling was, therefore, suitable as it was dependent on having the necessary information and experience of the phenomena being studied (Campbell et al., 2020). Target respondents were employees, HR professionals, recruiters, managers or members of an organisation with direct or indirect experience of AI-assisted recruitment and selection.

The following inclusion criteria were imposed: being currently employed, familiarity with recruitment or HR-related processes in their organisation, and exposure to AI recruitment systems either directly or indirectly. Direct exposure was defined as having utilised AI tools directly in recruitment processes, such as screening, shortlisting, communicating with candidates, or making decisions. Indirect exposure was defined as having seen, been trained in, interacted with or been impacted by AI in the organisation’s recruitment process. This difference was crucial as employee perceptions could be influenced by their direct experience with the AI system, as well as by organisational communication, perceived role implications, and experience with AI in their workplace. Study participation was made easy via online surveying through Google Forms across sectors/organisation categories. The introduction of the survey clearly stated that the focus was on AI in the recruitment and selection domain, and respondents were advised to only continue with the survey if they had some form of workplace exposure to AI.

Respondents were provided with the study purpose form and informed about participant rights and confidentiality protection on the consent form that they voluntarily signed. Research ethics guidelines were strictly followed, including the use of anonymous data and secure data storage. No personally identifiable information nor confidential data about organisations, proprietary recruitment tools or individual hiring decisions were collected. This was crucial because using AI for recruitment brings up a host of delicate topics, from fairness to professional judgement, employee trust, and accountability. The online survey format also helped to preserve confidentiality as respondents could fill out the survey without any direct pressure from the researchers. These procedures were used to ensure that respondents’ privacy was protected and the data were appropriate for aggregate statistical analysis.

### 5.3. Measures and Questionnaire Design

Multiple-item measures were adapted to fit the context of AI-enabled recruitment. Rather than relying on a single pre-existing instrument, items were adapted from several published sources and are presented in Appendix A. The measured constructs were transparency of AI, trust in AI systems, organisational support for AI use, perceived usefulness of AI, technostress, clarity of AI integration, perceived threat to professional judgement, perceived AI effectiveness, HR support and readiness, and employee perceptions of AI. As there are no definite guidelines for questionnaires, the five-point Likert scale was used, which is commonly used for structured attitudes and perceptions questionnaires (Joshi et al., 2015). The instrument was content reviewed by experts prior to data collection to determine the comprehensibility, context and conceptual consistency.

Being guided by a construct in organising the questionnaire enhances the clarity and decreases the ambiguity of the questionnaire. Transparency items measured the understanding of AI’s recruitment decisions, and trust items measured perceived fairness, reliability, and impartiality in regard to the AI’s recruitment decisions; organisational support items included training, managerial support, and institutional encouragement of AI’s use; and usefulness items included efficiency and quality of decisions made by AI. The strains and mental fatigue items on technostress were used to measure technostress, and the items on professional judgment were used to measure perceived threats to discretion and expertise. The purpose, process and role implications of the integration of AI were measured by clarity items, while the other scales measured perceived effectiveness, HR support/readiness and employee perceptions of AI. Item codes in the final CFA correspond to the order of items within each Appendix scale. The entire questionnaire was drafted in English as the target respondents were considered well-versed in the language.

### 5.4. Data Analysis Techniques

SPSS version 28 was used to calculate the summary statistics of demographic variables, test data normality, and conduct a preliminary review of item responses. Pre-SEM data screening was conducted on the completeness of data, code accuracy, missing values, outliers, and distributional assumptions. Cases with unusable or incomplete responses were not analysed, and cases with valid responses were used in the measurement and structural models. Preliminary reliability checks were performed before confirmatory model testing, and the items were tested using descriptive statistics (skewness and kurtosis) to determine abnormality. These actions were required since SEM requires a keen planning of the data before estimating the model and interpretating the outcomes (Kline, 2023). Analysis was carried out in AMOS software version 28, which provides the structural equation method in two steps (Sarstedt et al., 2022). The two-step process entailed the analysis of the measurement model followed by the analysis of relationships among the constructs.

Before interpreting the relationships among the measurement model, the model was evaluated. The internal consistency of the scales was examined using Cronbach’s alpha and composite reliability (CR); a value of around 0.70 or higher was considered acceptable based on the recommendations of (Kline, 2023) and (Hair et al., 2021). Convergent validity was determined by using average variance extracted (AVE) and 0.50 was set as the threshold for convergent validity (Fornell & Larcker, 1981; Hair et al., 2021). The Fornell–Larcker criterion was used to assess the discriminant validity of the constructs by comparing the square root of the AVE with the inter-construct correlations, and the heterotrait–monotrait ratio (HTMT) was used as an additional diagnostic to assess the discriminant validity (Fornell & Larcker, 1981; Roemer et al., 2021).

A number of fit indices for the structural model were computed: Root Mean Square Residual (RMR), Goodness-of-Fit Index (GFI), Adjusted Goodness-of-Fit Index (AGFI), Comparative Fit Index (CFI), Tucker–Lewis Index (TLI), the chi-square/degrees-of-freedom ratio (χ^2^/df), and Root Mean Square Error of Approximation (RMSEA). The separate CFA also reported SRMR. In the final AMOS structural specification, the seven exogenous latent constructs were freely covaried, and maximum-likelihood estimation converged successfully. Using several indices provides a more balanced assessment of model fit because individual statistics, particularly chi-square, can be sensitive to sample size (L. T. Hu & Bentler, 1999). Unstandardised (B) and standard errors (SE) were reported in addition to the critical ratio statistics, *p*-values and standardised coefficients (β) for the hypothesis tests. Indeed, the estimation of indirect effects is explicit in the SEM approach and is required in formal mediation. Perceived AI effectiveness and HR support/readiness were considered to be intermediate explanatory variables since indirect effects were not estimated and, therefore, were not considered statistical mediators.

### 5.5. Common Method Bias and Procedural Controls

Common method bias was accounted for in the research design, as well as in the interpretation of the self-reported data obtained from a cross-sectional survey. The questionnaire was designed in such a way that it reduced evaluation apprehension, ensuring anonymity, voluntary participation and confidentiality. The questions were phrased in a clear and neutral way. Furthermore, the online questionnaire was structured according to the investigated constructs of AI-enabled recruitment in order to avoid responses based on the participants’ impression. The predictor and outcome variables were gathered from the same source using the same instrument at the same point in time. Thus, common method variance had to be controlled by procedural means (Podsakoff et al., 2003). To reduce the chance that the relationships found in the study were mainly caused by social desirability, consistency motives, or the common scale format effects, procedural safeguards were incorporated.

Common method bias was considered because the predictor and outcome variables were collected from the same respondents using a single cross-sectional questionnaire. Procedural safeguards included anonymity, voluntary participation, neutral wording and the separation of construct-specific item blocks. Harman’s single-factor test was used only as a diagnostic because it cannot, by itself, establish the absence of common method variance (Podsakoff et al., 2003; Jordan & Troth, 2020). The first factor had an initial eigenvalue of 8.314, and nine factors had eigenvalues greater than 1.00. The single-source design, therefore, remained vulnerable to common method bias.

### 5.6. Ethical Considerations

This study followed the common ethical guidelines of surveys involving organisations. Participants filled out the questionnaire voluntarily and were informed about the intention of the study prior to completing the questionnaire. No personal information was sought, and all information collected was stored in a safe place. The questionnaire did not entice the participants to give confidential company information about particular AI systems, proprietary recruitment systems or hires. This allowed us to investigate the perceptions of employees towards AI-assisted recruitment at the corporate scale while ensuring participant privacy. Ethical issues were of special concern since AI recruitment involves sensitive issues, such as fairness, access to employment, professional judgement, and trust in company decision making.

## 6. Results

### 6.1. Data Screening and Preliminary Assessment

The dataset was screened before the main analysis to ensure that it was suitable for Structural Equation Modelling (SEM). The data screening process is shown in Table 1. It involved checking the completeness of the responses, consistency of coding, missing values, outliers, and distributional assumptions. The total number of valid responses for analysis was 450, after discarding unusable/incomplete responses. The current sample size was deemed to be sufficient for SEM, being greater than the minimum number of latent constructs and observed indicators that are often recommended. Normality of indicators was examined by looking at the skewness and kurtosis values, which were within acceptable ranges for covariance-based SEM. Sampling adequacy was also strong (KMO = 0.892), and Bartlett’s test of sphericity was significant, χ^2^(990) = 12,086.042, *p* < 0.001. Based on the preliminary assessment, AMOS 28 was then used for confirmatory factor analysis and testing of the structural model.

### 6.2. Common Method Bias Assessment

The common method bias was taken into account since all constructs were assessed with the help of the same cross-sectional self-report questionnaire (Podsakoff et al., 2003). Procedural safeguards were the anonymity, voluntary participation, and neutral wordings and separation of construct-specific item blocks. As a diagnostic, a one-factor test created by Harman was employed. As shown in Table 2, one of these factors had an eigenvalue of 8.314 and explained 18.48% of the total variance of the 45 items administered, with nine factors that had an eigenvalue greater than 1.00. The results did not suggest that there was a single dominant factor but, more than this, Harman’s test alone cannot rule out the possibility of common method bias (Podsakoff et al., 2003; Jordan & Troth, 2020). The results should, therefore, be interpreted cautiously.

### 6.3. Respondent Demographic Characteristics

The demographic results shown in Table 3 give an overview of the respondents who were included in the analysis. There were 450 cases from Jeddah, Riyadh and Dammam. The gender distribution was fairly even, with 240 male respondents (53.33%) and 210 female respondents (46.67%). This fairly even distribution of male and female employees allowed for analysis of employee perceptions regarding AI from a diverse organisational workforce.

With regard to employee type, non-government employees (286 respondents) represented the largest group (63.56%), while government employees (164 respondents) represented the second largest group (36.44%). This suggests greater representation from non-government organisations, although the presence of government staff meant that the research could reflect views on the issue of digital transformation in the public sector where there are more pronounced digital transformation initiatives. The age range was wide, and the largest group of respondents was in the 30–39 age group with 27.56%, followed by the 40–49 age group with 26.22%, the 20–29 age group with 24.44% and the 50 and above age group with 21.78%.

In general, the respondents were well educated, with the majority (54.22%) holding a bachelor’s degree, while 31.33% had a postgraduate degree, 10.00% were high school graduates and 4.44% were in the “Others” category. Geographically, there was an even distribution, with 155 respondents from Jeddah, 148 respondents from Riyadh and 147 respondents from Dammam. A balanced distribution across the three cities was achieved in order to strengthen the contextual coverage of this study, as these cities are major economic and technological centres in Saudi Arabia.

### 6.4. Measurement Model Assessment

The measurement model was tested first followed by the testing of the structural model. Standardised factor loadings, Cronbach’s alpha and composite reliability were used to assess the reliability of the indicator, while AVE was used to assess the convergent validity of the indicator (Kline, 2023; Fornell & Larcker, 1981; Hair et al., 2021). As shown in Table 4, all 45 items in the questionnaire were kept in the re-estimated CFA, and the standardised factor loadings were between 0.743 and 0.850. Thus, none of the indicators had to be deleted based on its loading. However, all items were retained as this was consistent with adequate construct coverage, as well as statistical performance (Hair et al., 2021). A comprehensive questionnaire that was kept in the CFA is given in Appendix A.

### 6.5. Internal Consistency and Convergent Validity

Cronbach’s alpha and composite reliability were used to determine internal consistency. All ten constructs showed strong reliability, with Cronbach’s alpha ranging from 0.854 to 0.909 and composite reliability ranging from 0.855 to 0.910. AVE values ranged from 0.595 to 0.670, exceeding the 0.50 threshold for every construct and supporting convergent validity. The results, therefore, supported the reliability and convergent validity of the measurement model, as presented in Table 5.

### 6.6. Discriminant Validity

Table 6 presents the square roots of the AVEs on the diagonal, consistent with the Fornell–Larcker criterion (Fornell & Larcker, 1981). All diagonal values exceeded the corresponding inter-construct correlations. The largest latent correlation was 0.555 between perceived AI effectiveness and employee perceptions of AI, below the square root of AVE for both constructs; therefore, no Fornell–Larcker discriminant validity concern was identified.

HTMT was additionally examined as a discriminant validity diagnostic (Roemer et al., 2021). The complete 45-pair HTMT matrix is reported in Table 7. Values ranged from 0.035 to 0.553, all below the 0.85 threshold. The highest value was 0.553 for perceived AI effectiveness–employee perceptions of AI, supporting discriminant validity across all ten constructs.

### 6.7. Structural Model Assessment

The explanatory pathways of the structural model were included in two ways. The first established a relationship between transparency of AI and trust in AI systems, as well as organisational support for AI use and perceived usefulness of the AI with the perceived effectiveness of the AI. The second one was related to technostress and perceived threats to professional judgement and clarity of AI integration with HR support and readiness. Next, perceived effectiveness of AI and HR level of support/readiness were linked to employees’ perceptions of the use of AI in recruitment and selection. Given the nature of cross-sectional data, which lack a time sequence, these paths were considered as theoretically defined structural relationship instead of causal relationships (Wang & Cheng, 2020).

The SEM results illustrated that transparency of AI, trust in AI systems, organizational support for using AI and perceived usefulness of AI were all significantly and positively related to perceived AI effectiveness. Perceived usefulness showed the strongest standardised effect (β = 0.421), followed by transparency (β = 0.309), trust (β = 0.279) and organisational support (β = 0.262). The model explained 51.5% of the variance in perceived AI effectiveness.

**Figure 2:**
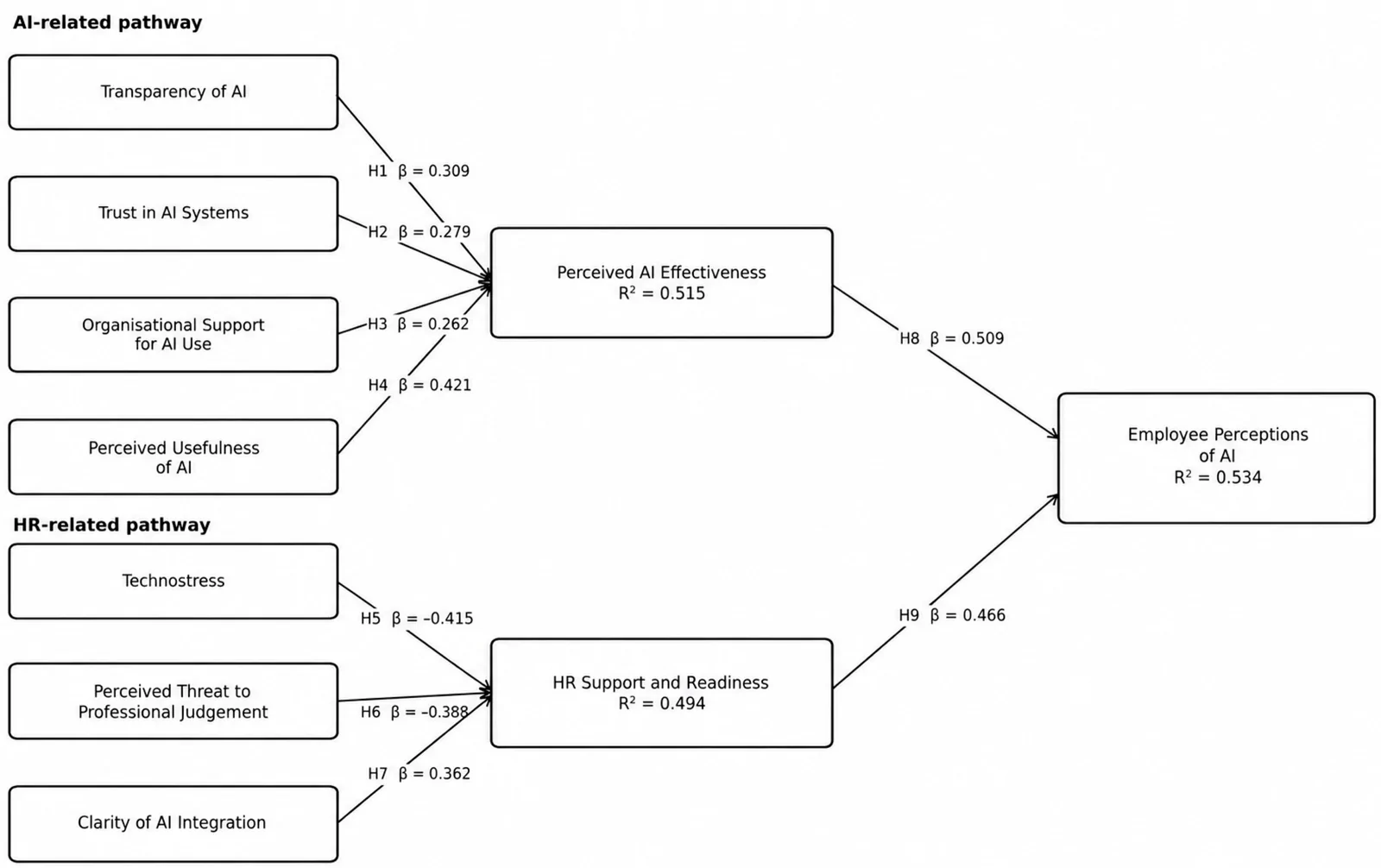
Final structural model with standardised coefficients and explained variance.

There were also significant associations in the HR pathway. Technostress (β = −0.415) and perceived threat to professional judgement (β = −0.388) were negatively associated with HR support and readiness, whereas clarity of AI integration was positively associated with HR support and readiness (β = 0.362). The model explained 49.4% of the variance in HR support/readiness. Perceived AI effectiveness (β = 0.509) and HR support/readiness (β = 0.466) were both positively related to the employees’ perception of AI, which accounted for 53.4% of the variance as presented in Figure 2. The collinearity diagnostics were low for all structural predictor sets, ranging from about 1.00 to 1.02, and did not pose a significant multicollinearity concern.

### 6.8. Model Fit Indices

Model fit was evaluated separately for the measurement and structural models (Table 8). The measurement model showed excellent fit, χ^2^(900) = 882.347, *p* = 0.657, CMIN/DF = 0.980, CFI = 1.000, TLI = 1.000, RMSEA = 0.000 and SRMR = 0.031. The final AMOS structural model also showed excellent fit, χ^2^(915) = 894.865, *p* = 0.677, CMIN/DF = 0.978, CFI = 1.000, TLI = 1.002 and RMSEA = 0.000.

The final AMOS solution reached the minimum successfully. AMOS additionally reported RMR = 0.048, GFI = 0.922, AGFI = 0.911, NFI = 0.929 and IFI = 1.002. The 90% confidence interval for RMSEA was [0.000, 0.011], with PCLOSE = 1.000. No post hoc correlated residuals or additional structural paths were introduced. Taken together, the fit statistics indicate that the structural specification represented the observed covariance structure well.

### 6.9. Hypothesis Testing

Hypothesis testing focused on the nine structural paths specified in the conceptual framework. Table 9 reports the AMOS maximum-likelihood unstandardised estimate (B), standard error (SE), critical ratio (CR), *p*-value and corresponding standardised coefficient (β). The first pathway estimates the four AI-related antecedents of perceived AI effectiveness; the second estimates the three HR-related antecedents of HR support/readiness; the final two paths estimate employee perceptions of AI.

Positive results were obtained from the test of the two end result paths, between the end result paths and employee perceptions of AI in recruitment and selection, and between the two end result paths and employee perceptions of HR in recruitment and selection. Positive relationships were found between transparency and employees’ rating of the perceived effectiveness of AI systems, i.e., the transparency of AI systems was found to be positively related to employees’ rating of the effectiveness of AI systems used in recruitment and selection; the more transparent the AI systems used in recruitment and selection, the higher the rating by employees regarding the effectiveness of these systems.

Trust in AI was positive in predicting perceived AI effectiveness, suggesting that trust in the fairness, reliability and accountability of AI was positively associated with effectiveness ratings of the technology. Organisational support was also positively related to perceived effectiveness, indicating that training, managerial encouragement and implementation support helped to positively evaluate AI. The highest coefficient was for perceived usefulness, suggesting that employees’ evaluation of the effectiveness of AI was most strongly associated with perceived usefulness in recruitment.

The HR support/readiness pathway was also supported. The relationship between technostress and HR support and readiness was found to be negative, which means that the higher the reported technostress levels, the lower the perceived readiness of the HR environment (HR support). Finally, a negative correlation between perceived threat to professional judgement and HR support and readiness was found, suggesting that a decrease in perceived autonomy or perceived devaluation of human skills was associated with a decrease in perceived HR support and readiness. Clarity of AI integration was positively associated with HR support and readiness, suggesting that employees sensed that HR was more prepared when they grasped the purpose, scope and implications to their roles when it came to AI implementation.

Perceived AI effectiveness and HR support/readiness were both positive predictors of employee perceptions of AI in the final outcome paths. The standardised coefficient on perceived AI effectiveness was larger, which means that the relationship between this factor and the perceptions of employees is stronger than the relationship between this factor and HR support/readiness. HR support and readiness, however, were also a significant positive predictor, which indicated that positive perceptions of AI were related to both system capability and positive organisational climate. The structural model explained 51.5% of the variance in perceived AI effectiveness, 49.4% in HR support/readiness and 53.4% in employee perceptions of AI. Overall, the findings agree with the conceptual model, indicating that employee perceptions of AI recruitment can be influenced by both technical and socio-technical routes.

### 6.10. Summary of Results

The preliminary assessment indicated that the dataset was suitable for SEM, with KMO = 0.892 and a significant Bartlett’s test. The sample comprised 450 respondents from Jeddah, Riyadh and Dammam. All 45 indicators were retained in the CFA, standardised loadings ranged from 0.743 to 0.850, and all constructs met reliability and convergent-validity criteria. The complete HTMT matrix contained 45 pairwise comparisons, with a maximum value of 0.553, while the Fornell–Larcker criterion was satisfied for every construct. The measurement and structural models both showed excellent fit. Transparency, trust, organisational support and perceived usefulness were positively associated with perceived AI effectiveness; technostress and perceived threat to professional judgement were negatively associated with HR support/readiness, whereas clarity of AI integration was positively associated with it. Perceived AI effectiveness and HR support/readiness were both positively associated with employee perceptions of AI. The model explained 51.5%, 49.4% and 53.4% of the variance in perceived AI effectiveness, HR support/readiness and employee perceptions of AI, respectively. Due to the design of a cross-sectional study, the results should be viewed as correlations in the two-path model, as proposed by Wang and Cheng (2020).

## 7. Discussions, Implications, Limitations, and Future Research

### 7.1. Discussion and Implications

#### 7.1.1. AI-Related Pathway and Employee Perceptions of AI

The results indicated that perceived AI effectiveness had a positive relationship with transparency, trust, organisational support and perceived usefulness and was also positively linked with employee perceptions of AI. The perceived usefulness was the most predictive in the AI effectiveness pathway, in line with the results that perceived performance benefits are at the core of willingness to use AI among employees (Almeida et al., 2025; Ibrahim et al., 2025). Perceived AI effectiveness was also positively related to trust, as expected, as research also pointed to trust as a necessary factor when accepting AI systems (Afroogh et al., 2024; Y. Xu et al., 2024). Transparency was positively related to perceived AI effectiveness, and it can be explained by findings that explainability and visibility can enhance trust and judgments of AI-backed decisions (Employees’ appraisals and trust of artificial intelligences’ transparency and opacity, Yu et al., 2023; Artificial intelligence decision-making transparency and employees’ trust: The parallel multiple mediating effect of effectiveness and discomfort, Yu & Li, 2022). Organisational support likewise played a positive role to boost the research that highlights training, managerial support and implementation resources as enabling factors to AI adoption (C. Hu et al., 2025; R. B. Soomro et al., 2025). In general, the perceived system capability and organisational assurance seem to be reflected in employee ratings of AI recruitment.

#### 7.1.2. HR-Related Pathway and Employee Perceptions of AI

The HR pathway exhibited a variant. On the one hand, the factors of HR support and readiness were negatively related to the technostress and perceived threat to professional judgement, whereas the clarity of AI integration was positively related to HR support and readiness. This trend is aligned with the results of socio-technical research, demonstrating that AI-related stress may turn into a liability when technological requirements seem to be hard to cope with (Jin et al., 2026). Such associations are not to be construed as causations since supportive policies, training and HR support might precede and minimize technostress or the perceived threat to the profession, and cross-sectional data cannot determine the chronological order (Wang & Cheng, 2020). Studies of human–AI collaboration also indicate that intelligent technologies can produce resource-enhancing and resource-straining evaluations of employees (AI collaboration and quality of work life: a dual perspective of hotel employees’ perceptions, Duan et al., 2026b). The HR route, thus, indicates reflective, supportive and strain-related circumstances around AI implementation.

#### 7.1.3. Implications

The broader implication is that the introduction of AI must be handled with a dual emphasis on the capacity of the systems and socio-technical preparedness. Positive predictors such as utility and transparency imply that, when an AI system is well designed and perceived as beneficial, employees will tend to react better. The role of organisational support, however, implies that the focus should be on institutional preparedness, as managers should be conscious and proactively control and facilitate the integration of the tools that they implement. S. Soomro et al. (2024) considered managerial commitment to have a buffer role against anxiety and scepticism, which is in line with the above finding. Companies that publicise how they implement AI in their systems, e.g., by publishing data and information on privacy concerns, are more likely to engender trust and positive attitudes among employees (Gong et al., 2024; Khan et al., 2024). The negative correlation between technostress and role threat suggests that there is a need for early education and interventions to increase frontline employees’ understanding of how AI can be used to enhance their control, confidence, and relevance in the recruitment process. AI is more likely to be resisted if it is introduced as a substitute for human workers, not as a tool to help them (Chang et al., 2024; Gardes, 2025). Thus, organisations must incorporate relational procedures such as training, co-creation and transparency into their design principles and deployment procedures. They can make their AI take-up sustainable and comprehensive by taking into consideration both system capability and human aspects.

The findings support the use of UTAUT and Socio-Technical Theory as complementary explanatory lenses rather than as a single causal sequence. The AI effectiveness pathway reflects technology acceptance mechanisms related to perceived usefulness, system trust, transparency and facilitating support (Venkatesh et al., 2003; Dwivedi et al., 2019). The HR support/readiness pathway reflects the socio-technical interaction between technological change, work roles, psychological strain and organisational support (Trist & Bamforth, 1951; Pasmore, 1985). Together, the two perspectives explain employee perceptions of AI through both technical evaluations of the system and the social and organisational conditions surrounding implementation. This interpretation is consistent with the cross-sectional design and does not imply temporal causality.

### 7.2. Limitations

There are several limitations that should be considered when interpreting the findings. First, the cross-sectional design used in this study does not allow for temporal order or causality to be established and is susceptible to common method bias (Podsakoff et al., 2003; Jordan & Troth, 2020; Wang & Cheng, 2020). The single-factor test by Harman is not extensive as it is a diagnostic test that is unable to rule out common method variance (Podsakoff et al., 2003; Jordan & Troth, 2020). Secondly, the respondents consisted of HR professionals, recruiters, and managers or other staff members who are directly or indirectly involved in the recruitment process with AI tools. Measurement invariance and multi-group structural differences were not tested, although these groups may have different interpretations of the measurement of work efficiency, professional judgment, and items specific to recruitment. Third, it was not possible to determine the direction of the relationships between technostress and professional threat and HR support/readiness due to the fact that there may be reverse or reciprocal relationships in cross-sectional data (Wang & Cheng, 2020). All pairs of constructs were found to support complete discriminant validity for the HTMT (Roemer et al., 2021) with the exception of some that could be replicated with independent samples. Longitudinal designs and multi-group analysis and behavioural measures are recommended for future research to enhance temporal and measurement inferences.

### 7.3. Conclusions and Future Research

The study revealed positive relationships between perceived usefulness, employee perceptions of AI in recruitment, trust, organisational support, clarity and transparency, as well as negative relationships between employee perceptions of AI in recruitment and technostress and perceived threat to professional judgement. These results need to be explained by two explanatory pathways: AI-related factors and the perceived effectiveness of AI (pathway 1), and HR-related factors and HR support and readiness (pathway 2). Both intermediate factors are associated with employee perceptions of AI. The results are not a formal test of statistical mediation since indirect effects were not formally tested. In general, the employee attitudes towards AI recruitment seem to reveal the socio-technical context of AI implementation and evaluations of AI capabilities.

Longitudinal studies are needed to explore the attitude changes of employees with the passage of time and at different phases of AI deployment. Further, qualitative approaches (interviews or ethnographic studies) might give a better understanding of the emotional, cultural and organisational dimensions influencing the acceptance of AI. Cross-cultural comparisons and variations by sector can be examined to determine significant contextual factors, especially in sectors where automation is actively developing. Finally, further research is needed on how leadership styles, forms of governance and employee engagement influence the design of AI and the ensuing trust and resistance and subsequent adoption.

The impact of actual AI exposure on employees in the long term should also be explored in future research. There may be differences in perceptions of usefulness, trust, and stress between employees who directly use AI recruitment tools and those who are merely hearing about or witnessing the use of AI. Subsequent studies can compare direct users, indirect users, HR professionals, line managers, and applicants in terms of whether there is a disconnect in how AI is perceived by these different stakeholder groups. It would also be worthwhile to use a mixed-methods approach since interviews may deepen our understanding of why employees trust or distrust AI, and resist or reinterpret AI, which may not be apparent from survey responses. These extensions would further help to explain the process of acceptance, contestation and normalisation of AI recruitment in organisational practice.

## Figures and Tables

**Table 1:** Summary of data screening process.

Screening Criterion	Result	Decision
Initial responses received	461	Suitable
Invalid/incomplete responses removed	11	Excluded
Final valid responses	450	Retained
Missing item responses	0	Acceptable
Multivariate outliers	0 at *p* < 0.001	Acceptable
Item normality	Max |skew| = 0.177; max |excess kurtosis| = 1.086	Acceptable for SEM
KMO sampling adequacy	0.892	Good
Bartlett’s test of sphericity	χ^2^(990) = 12,086.042, *p* < 0.001	Suitable for factor analysis
Final dataset for SEM	450	Suitable

**Table 2:** Harman’s single-factor test for common method bias.

Interpretation	Result	Criterion	Test
No dominant single factor	18.48%	<50%	First unrotated factor variance
First component retained	8.314	>1 retained	First-factor eigenvalue
Multiple factors retained	9	>1 expected	Factors with eigenvalue > 1
CMB not ruled out	No dominant factor	Diagnostic only	Overall interpretation

**Table 3:** Distribution of respondents by demographic characteristics.

Gender			Employee Type		
Female	210	46.67%	Government	164	36.44%
Male	240	53.33%	Non-Government	286	63.56%
	450	100.00%		450	100.00%
Age			Education		
20–29	110	24.44%	High School	45	10.00%
30–39	124	27.56%	Bachelor’s Degree	244	54.22%
40–49	118	26.22%	Post-Graduate	141	31.33%
50+	98	21.78%	Others	20	4.44%
	450	100.00%		450	100.00%
City					
Jeddah	155	34.44%			
Riyadh	148	32.89%			
Dammam	147	32.67%			
	450	100.00%			

**Table 4:** Standardised factor loadings.

Construct	Item	Standardised Loading	Decision
Trust in AI Systems	TRST1	0.833	Retained
	TRST2	0.809	Retained
	TRST3	0.803	Retained
	TRST4	0.824	Retained
Organisational Support for AI Use	SPRT1	0.836	Retained
	SPRT2	0.802	Retained
	SPRT3	0.800	Retained
	SPRT4	0.817	Retained
	SPRT5	0.780	Retained
Transparency of AI	TRSP1	0.839	Retained
	TRSP2	0.830	Retained
	TRSP3	0.744	Retained
	TRSP4	0.834	Retained
	TRSP5	0.824	Retained
Perceived Usefulness of AI	USFL1	0.779	Retained
	USFL2	0.780	Retained
	USFL3	0.796	Retained
	USFL4	0.782	Retained
Technostress	TSTR1	0.792	Retained
	TSTR2	0.795	Retained
	TSTR3	0.808	Retained
	TSTR4	0.821	Retained
Clarity of AI Integration	CLINT1	0.789	Retained
	CLINT2	0.807	Retained
	CLINT3	0.746	Retained
	CLINT4	0.821	Retained
	CLINT5	0.778	Retained
Perceived Threat to Professional Judgement	THRT1	0.762	Retained
	THRT2	0.802	Retained
	THRT3	0.803	Retained
	THRT4	0.779	Retained
	THRT5	0.815	Retained
HR Support and Readiness	HRSUP1	0.753	Retained
	HRSUP2	0.743	Retained
	HRSUP3	0.795	Retained
	HRSUP4	0.794	Retained
Perceived AI Effectiveness	AIEFF1	0.794	Retained
	AIEFF2	0.785	Retained
	AIEFF3	0.802	Retained
	AIEFF4	0.799	Retained
Employee Perceptions of AI	PCPTN1	0.770	Retained
	PCPTN2	0.849	Retained
	PCPTN3	0.802	Retained
	PCPTN4	0.819	Retained
	PCPTN5	0.850	Retained

**Table 5:** Reliability and convergent validity results.

Construct	No. of Items	Cronbach’s Alpha	Composite Reliability	AVE	Decision
Trust in AI Systems	4	0.889	0.890	0.668	Acceptable
Organisational Support for AI Use	5	0.903	0.903	0.652	Acceptable
Transparency of AI	5	0.908	0.908	0.664	Acceptable
Perceived Usefulness of AI	4	0.865	0.865	0.615	Acceptable
Technostress	4	0.880	0.880	0.647	Acceptable
Clarity of AI Integration	5	0.891	0.891	0.622	Acceptable
Perceived Threat to Professional Judgement	5	0.894	0.894	0.628	Acceptable
HR Support and Readiness	4	0.854	0.855	0.595	Acceptable
Perceived AI Effectiveness	4	0.873	0.873	0.632	Acceptable
Employee Perceptions of AI	5	0.909	0.910	0.670	Acceptable

**Table 6:** Fornell–Larcker discriminant validity matrix.

Construct	TRST	SPRT	TRSP	USFL	TSTR	CLINT	THRT	HR	AI	PCPTN
TRST	0.817									
SPRT	0.055	0.807								
TRSP	0.083	0.083	0.815							
USFL	0.105	0.084	0.062	0.784						
TSTR	−0.112	0.024	−0.044	−0.040	0.804					
CLINT	0.143	0.042	0.145	0.200	−0.072	0.789				
THRT	−0.111	0.001	−0.116	−0.058	−0.016	−0.082	0.792			
HR	0.130	−0.025	0.162	0.079	−0.438	0.428	−0.410	0.771		
AI	0.359	0.343	0.383	0.499	−0.087	0.157	−0.032	0.067	0.795	
PCPTN	0.294	0.133	0.253	0.244	−0.224	0.256	−0.223	0.515	0.555	0.819

**Table 7:** Complete HTMT discriminant validity matrix.

Construct	TRST	SPRT	TRSP	USFL	TSTR	CLINT	THRT	HR	AI	PCPTN
TRST	—									
SPRT	0.061	—								
TRSP	0.081	0.078	—							
USFL	0.108	0.086	0.066	—						
TSTR	0.116	0.037	0.056	0.070	—					
CLINT	0.141	0.067	0.147	0.202	0.076	—				
THRT	0.114	0.035	0.116	0.060	0.048	0.086	—			
HR	0.127	0.042	0.164	0.083	0.437	0.429	0.407	—		
AI	0.357	0.342	0.376	0.499	0.088	0.162	0.042	0.068	—	
PCPTN	0.290	0.137	0.249	0.240	0.228	0.259	0.223	0.514	0.553	—

**Table 8:** Measurement and structural model fit indices.

Fit Index	Recommended Guideline	Measurement Model	Structural Model	Interpretation
χ^2^/CMIN	—	882.347	894.865	Reported
Degrees of freedom	—	900	915	Reported
*p*-value	*p* > 0.05 desirable	0.657	0.677	Good fit
CMIN/DF	<3.00	0.980	0.978	Good fit
CFI	≥0.90 acceptable; ≥0.95 good	1.000	1.000	Good fit
TLI	≥0.90 acceptable; ≥0.95 good	1.000	1.002	Good fit
RMSEA	≤0.08 acceptable; ≤0.06 good	0.000	0.000	Good fit
RMSEA 90% CI	Narrow interval preferred	—	[0.000, 0.011]	Good fit
PCLOSE	>0.05	—	1.000	Good fit
RMR	Lower values preferred	—	0.048	Low residual
GFI	≥0.90	—	0.922	Acceptable
AGFI	≥0.90	—	0.911	Acceptable
NFI	≥0.90	—	0.929	Acceptable
IFI	≥0.90	—	1.002	Good fit

**Table 9:** Hypothesis testing results (unstandardised and standardised estimates).

**H**	Structural Path	B	SE	CR	*p*	Std. β	Decision
H1	Transparency → Perceived AI Effectiveness	0.280	0.039	7.106	<0.001	0.309	Supported
H2	Trust in AI → Perceived AI Effectiveness	0.261	0.041	6.409	<0.001	0.279	Supported
H3	Organisational Support → Perceived AI Effectiveness	0.247	0.040	6.130	<0.001	0.262	Supported
H4	Perceived Usefulness → Perceived AI Effectiveness	0.444	0.050	8.857	<0.001	0.421	Supported
H5	Technostress → HR Support/Readiness	−0.400	0.046	−8.701	<0.001	−0.415	Supported
H6	Professional-Judgement Threat → HR Support/Readiness	−0.377	0.045	−8.356	<0.001	−0.388	Supported
H7	AI-Integration Clarity → HR Support/Readiness	0.379	0.049	7.665	<0.001	0.362	Supported
H8	Perceived AI Effectiveness → Employee Perceptions of AI	0.565	0.051	11.149	<0.001	0.509	Supported
H9	HR Support/Readiness → Employee Perceptions of AI	0.528	0.051	10.268	<0.001	0.466	Supported

## Data Availability

The datasets generated during and/or analysed during the current study are not publicly available due to data protection and the policy of the ethics committee but are available from the author on reasonable request.

## Appendix A. Survey Questionnaire

**Table A1:** Transparency of AI.

Question	Source
The system explicitly outlines the way recruitment choices are determined.	(Employees’ appraisals and trust of artificial intelligences’ transparency and opacity, Yu et al., 2023)
I know the information the AI system relies upon when considering candidates.	(Artificial intelligence decision-making transparency and employees’ trust: The parallel multiple mediating effect of effectiveness and discomfort, Yu & Li, 2022)
The process of the AI hiring system making a choice is transparent.	(Kovács & Horváth, 2025)
I comprehend the reasoning behind the recommendations of the AI system.	(Artificial intelligence decision-making transparency and employees’ trust: The parallel multiple mediating effect of effectiveness and discomfort, Yu & Li, 2022)
The tool gives adequate feedback upon the evaluations conducted.	(Horodyski, 2023)

**Table A2:** Trust in AI systems.

Question	Source
I have confidence in the hiring choices made by the AI system.	(Afroogh et al., 2024)
The system fairly judges candidates while shortlisting them.	(Y. Xu et al., 2024)
I trust using the advice of AI when making hiring choices.	(Kovács & Horváth, 2025)
I think the AI system remains impartial in its evaluations.	(Ackerhans et al., 2025)

**Table A3:** Organisational support for AI use.

Question	Source
We promote the utilisation of AI when it comes to recruitment.	(C. Hu et al., 2025)
There is proper training in the use of AI-based recruitment tools.	(G. Xu et al., 2023)
Management supplies support to assist with AI-driven hiring.	(S. Soomro et al., 2024)
I feel supported while adjusting to new work-related AI technologies	(Gardes, 2025)
My organisation values employee input on AI system usage.	(Sadeghi, 2024)

**Table A4:** Perceived usefulness of AI.

Question	Source
AI enhances the process efficiency in recruitment.	(Alsaedi et al., 2024)
Utilising AI in hiring facilitates more accurate hiring choices.	(G. Xu et al., 2023)
AI saves time over the usual ways of hiring.	(Alsalman, 2025)
AI enhances the value of the entire HR function.	(Sadeghi, 2024)

**Table A5:** Technostress.

Question	Source
I get overwhelmed when utilising recruitment AI tools.	(Horodyski, 2023)
I get stressed when attempting to comprehend the way AI systems operate.	(Kim & Lee, 2024)
The implementation of AI places increased pressure on my workload.	(Chang et al., 2024)
Adapting to the use of AI in the HR department mentally tires me out.	(Horodyski, 2023)

**Table A6:** Perceived threat to human judgement.

Question	Source
Artificial intelligence in recruitment puts human judgement in jeopardy.	(Golgeci et al., 2025)
I believe my experience is being devalued when hiring incorporates the use of AI.	(Ackerhans et al., 2025)
The HR professionals’ work can potentially be replaced by the AI system.	(Gardes, 2025)
I worry that HR professionals will become outdated.	(Alsalman, 2025)
AI diminishes my role in important recruitment choices.	(Horodyski, 2023)

**Table A7:** Clarity of AI integration.

Question	Source
The reasons for deploying AI technologies within our company have been well explained.	(Mwita & Kitole, 2025)
I have a solid grasp of how AI integration supports our organisational goals.	(Khan et al., 2024)
Details supplied regarding steps for AI implementation prove transparent and straightforward.	(Gong et al., 2024)
I comprehend the anticipated adjustments in my job as a result of AI integration.	(Gong et al., 2024)
The company has well-established procedures for managing issues resulting from AI integration.	(Khan et al., 2024)

**Table A8:** AI effectiveness.

Question	Source
AI tools have improved my overall work efficiency.	(Almeida et al., 2025)
The output of AI systems aligns with our organisational goals.	(Köchling et al., 2025)
AI has enhanced the quality of decision-making in my role.	(Kovács & Horváth, 2025)
Using AI has reduced the time needed to complete complex tasks.	(Sadeghi, 2024)

**Table A9:** Human resource support.

Question	Source
My HR department provides sufficient training on AI tools.	(Tursunbayeva et al., 2025)
HR policies support the integration of AI in daily tasks.	(Cai et al., 2024)
I can rely on HR for help when facing AI-related challenges.	(Cai et al., 2024)
The HR team ensures fair AI implementation across departments.	(Cai et al., 2024)

**Table A10:** Employee perceptions.

Question	Source
I maintain a positive general impression of AI in hiring.	(Horodyski, 2023)
I think AI adds value to our hiring process.	(Horodyski, 2023)
Artificial intelligence has enhanced my profession as a recruitment expert.	(Sadeghi, 2024)
I am comfortable with using AI technologies.	(Sadeghi, 2024)
I am willing to consider increased AI use in hiring.	(Horodyski, 2023)
